# Emergent Crisis of COVID-19 Pandemic: Mental Health Challenges and Opportunities

**DOI:** 10.3389/fpsyt.2021.631008

**Published:** 2021-07-19

**Authors:** Amir Radfar, Maria M. Ferreira, Juan P. Sosa, Irina Filip

**Affiliations:** ^1^Assistant Professor of Medical Education, University of Central Florida, Orlando, FL, United States; ^2^Division of Research, Universidad Autónoma de Bucaramanga, Bucaramanga, Colombia; ^3^Division of Research, Universidad Nacional de Tucuman, San Miguel de Tucumán, Argentina; ^4^Department of Clinical Psychiatry, Western University of Health Sciences, Pomona, CA, United States

**Keywords:** mental health services, mental health, COVID-19, pandemic, economic crisis, policy development, public health

## Abstract

Mental health is a fundamental human right and is part of the well-being of society. The public health burden of mental health disorders affects people's social and economic status around the world. Coronavirus's (COVID-19) negative impact on the economy and mental health worldwide is concerning. This is a worldwide emergency, and there is an urgent need for research about this topic to prevent long-lasting adverse effects on the population. Unpreparedness and inconsistencies in guidelines, lockdowns, containment strategies, unemployment, financial losses, physical distancing, isolation, chaos, and uncertainty are among factors that lead to a rise in emotional distress, anxiety, and depression. Governments' decisions affect the socioeconomic status of a country and the psychological well-being of the people. COVID-19 pandemic exposed disparities in multiple mental health care systems by having adverse mental health effects in people with pre-existing mental health disorders and previously healthy individuals. Aggregation of concurrent or cumulative comorbid risk factors for COVID-19 disease and its psychosocial sequelae could provide invaluable information for the public health stakeholders. This review aims to address the burden and the psychosocial impact of the COVID-19 pandemic, the challenges and opportunities facing mental health systems, and proposes new strategies to improve the mental health outcomes in the post-COVID era.

## Introduction

### The Burden of Mental Disorders

Public Health is a fundamental human right, and governments are responsible for providing a healthy environment for the population. Mental health is part of public health and is defined by the World Health Organization (WHO) as a “state of wellbeing in which the individual realizes his or her own abilities can cope with the normal stresses of life, can work productively and fruitfully and is able to make a contribution to his or her community” (1). Public health systems are influential in providing, maintaining, shaping mental health services, and designing policies for the mentally ill and the population at risk.

The mental and addictive disorders' point prevalence worldwide in 2016 was 1,110,075,000 (16% of the world's population) (2). Mental and addictive disorders caused the loss of 162.5 million Disability-Adjusted Life Years (DALYs) and comprised 7% of all global burden of disease as measured in DALYs and 19% of all global Years Lived with Disability (YLD) in 2016 (2).

Based on the Global Burden of Disease study's findings, poor mental health and substance use disorders increased by 11% in the U.S. between 1990 and 2016 (3). Mental health issues are among the top 10 causes of premature death and disability in males and females in 2016 (3).

Additionally, it is currently one of the most expensive health care issues, affecting the health care industry, lowering productivity, and increasing costs in the U.S. economy (4). United States indicators estimate that 47.6 million adults aged 18 or older (19.1 %) had any mental illness, 11.4 million adults (23.9%) had a severe mental illness (SMI) and 3.5 million adolescents (14.4%) had a major depressive episode in 2018 (5).

There is also a geographical variation and socio-demographic status in the U.S.'s prevalence of mental health problems (6). East South-Central U.S. had the highest prevalence rate, with 14.88%, and West North-Central had the lowest rate, with 9.42% (6).

Mental disorders also affect social stability and reduce the quality of life. This leads to low educational attainment, decreases motivation and performance, impairment in personal and family functioning, discrimination, low income, increased poverty, violence, and higher mortality and suicide rates (7). The consequences of non-treatment increase the likelihood of violent and aggressive behaviors. Individuals with untreated or partially treated schizophrenia and bipolar disorder commit about 10% of all homicides in the U.S. and 33% of mass killings (8). Those with severe mental illness have 11 times increased likelihood of being victims of violence, assault, rape, or robbery (9). One-third of the homeless population is made up of individuals with an untreated mental illness (10). About 20% of jail inmates and 15% in state prisons have a severe mental illness (11). Failure to provide adequate care to patients with mental illness can turn into a social disaster.

Although the understanding of mental problems has evolved in the past decades, America's mental health system crisis has been a problem for many years. Despite all efforts made to deliver high-quality mental care, there are still gaps that need to be addressed. A 2018 survey from the National Council on Behavioral Health (NCBH) showed that 56% of patients want to access a mental healthcare provider, but many face barriers to care (12). Delivery of mental health care is determined by the financial resources available and has been a responsibility of the states, government insurances (Medicare and Medicaid), private providers (private insurance and out-of-pocket), and NGOs.

This review tries to shed light on the status of the mental health post-COVID-19 era, underscores the U.S. mental health care system's shortages, and proposes strategies and opportunities to improve mental health outcomes.

## Discussion

### COVID-19 Pandemic and Mental Health Issues

Past tragedies have shown long-lasting consequences on mental health and could contribute to a greater prevalence of mental disorders than the pandemic itself (13). It has been reported that the rates of suicide may momentarily decrease immediately after the initial disaster period, and inversely followed by a consequent increase in suicidal behaviors (14). Vulnerable populations such as people with mental health problems incarcerated population, victims of sexual and physical violence, the bereaved, minorities such as lesbian, gay, bisexual, transgender, and queer (LGBTQ) community are especially at risk.

COVID-19 pandemic can lead to a secondary mental illness paradigm. The pandemic's burden on mental health triggered an economic crisis by posing an increased risk of suicide and long-standing emotional distress. The economic decline due to unemployment, financial worries, increased isolation and lack of support, increased strain and violence in relationships due to confinement at home, decreased contact with people from outside the home who can provide support, decreased access to mental health services are among factors contributing to the increased risk of psychological distress and suicide and highlights the need and necessities of mental health programs such as suicide prevention programs (15). A web-based survey conducted post-COVID-19 outbreak in China shows that young individuals are more prone to developing depressive and generalized anxiety disorders and lower sleep quality than older people. The study found that younger people with a higher time spent on social media and healthcare workers overthinking about the outbreak were at high risk of mental illness (16). Fear of infection, anxiety, anger, post-traumatic stress disorder, stigma, avoidant behaviors, boredom, and frustration could contribute to individuals' mental health post COVID. The hypochondriac concerns add to other psychosocial and economic stressors, limited socialization, and isolation problems (17).

As the COVID-19 pandemic brings gaps in mental health services back to attention, it raises additional concerns. Prior studies conducted during the HIN1 and SARS outbreaks emphasize the mental health burden on health professionals. Feelings of uncertainty, vulnerability, fear of death, irritability, psychological distress, restriction of social contacts, and intentional absenteeism were seen in quarantined medical staff and are anticipated to be seen during the COVID-19 pandemic (18, 19).

Mental health care gaps between the need for treatment and the available services always existed but were amplified by the COVID-19 pandemic and became a worldwide emergency.

Recent findings reveal substantial neuropsychiatric morbidities in the 6 months after COVID-19 infection. These risks were higher in, but not limited to, patients who had severe COVID-19 (20).

Some of these challenges include limited access to care, limited availability and affordability of mental health care services, lack of funding, high priced drugs, lack of psychiatric beds, insurance and policies gaps, physician shortage, unintegrated system, treatment gaps, insufficient mental health care policies, stigma, post-COVID-19 syndrome, and lack of education on mental illness (21).

## Mental Health Care in United States And Its Chalenges

In the United States, based on the U.S. Constitution and the U.S. federalist system, the federal and state governments are responsible for their citizens' mental health. They are responsible for developing mental health policies and setting standards that effectively allow the public and private sectors to deliver mental health care, which can influence shaping services and policies for mentally ill patients.

The federal government participates in developing laws and regulations of mental health systems and providers; they also play a role in protecting individuals' rights with mental health disorders and supporting and funding services, research, and innovation (35). On the other hand, the states' mental health system must meet specific federal government standards since they have significant power in making decisions to expand beyond what exists at the federal level and improve services, access, and protections for consumers (36).

### Mental Health Financial Budget Shortages

The financing of mental health services changed dramatically over the years. The U.S. government allocated about $723 million for mental health services, $150 million for community behavioral health centers, $125 million for children's mental health services, and $133 million for school violence prevention in 2020 (37). The U.S. government's Fiscal Year 2020 budget revealed critical shortages and cutbacks for Medicaid, Medicare, and mental health research, putting at risk the quality of mental health care (22). This fund reduction created more gaps in the mental health system.

Public mental health services significantly deteriorated over the past three decades, suggesting that a fair amount of mental health care funds is lost to fraud, excess profits, or are wasted, rather than being used toward mental health care (38). With Medicare (national health insurance programs in the United States) being the largest source of funds for institutionalized patients and lacking incentives to finance a more extended inpatient stay, patients continue to be discharged from hospitals to live on the streets ending up being homeless or in jails. In addition, for the fiscal year 2021, National Tobacco Control Program, Pediatric Mental Healthcare Access grants, children's hospitals graduate medical education (CHGME) budgets either eliminated or flat-funded from previous year levels (22).

### Physicians, Mental Health Care Providers Shortage and Treatment Gaps

The gaps in treatment, diagnosis, and knowledge of mental disorders are a worldwide and public health problem and priority. Common treatment barriers of the psychiatric population are lack or difficulty in accessing mental health care, physician shortage, increasing prevalence and incidence of mental disorders, lack of infrastructure and psychiatric beds, financial gaps, rising drug pricing, lack of insurance, and more.

The federal and states governments provide many supportive programs to assist individuals with mental illnesses, and it connects them with job placement services, housing, behavioral health, and housing to divert them from the criminal justice system. However, these agencies do not specifically focus on assisting individuals with serious mental illness, which has led to fragmentation among these programs due to a lack of coordination among agencies (23).

According to The Substance Abuse and Mental Health Services Administration (SAMHSA), nearly 91 million Americans live in regions with severe shortages in available mental health professionals and estimates that a minimum of 1,846 psychiatrists and 5,931 other practitioners would be necessary to fill these gaps (24). The Association of American Medical Colleges projects that the United States will see a shortage of up to 122,000 physicians by 2032, with demands exceeding supply not only in primary care but also among specialist physicians (25).

In addition, there is an increase in severe mental illnesses among young adults (18–25 years old) and adults (26–49 years old) in 2018 (39). About 46.5% of young adults and 36.3% of adults were untreated in 2018 (39).

The Government Accountability Office reported 112 federal programs supporting individuals with serious mental illness and found a lack of interagency coordination and a lack of completed program evaluations (23). The uncoordinated agencies and care delivery, the lack of monitoring and assessment of the efficacy of programs, stigma, and the failure to detect and treat mental health conditions create gaps in the mental health care system leading to failure to meet the needs of this vulnerable population. Furthermore, new policies seeking the repealing and replacement of the Patient Protection and Affordable Care Act (ACA), which were developed since creating the American Health Care Act (AHCA) in 2017, lead to new disparities and limitations in mental health care and substance abuse disorders, affecting the quality and access to mental health services.

Moreover, even though more interventions and drugs are available every year, not everyone will have access to them due to the rising costs. The drug pricing system is a highly complex process that involves manufacturers, states, wholesalers, and pharmacies. The more significant limitation of this system is that there is no direct transaction between manufacturers and patients. Instead, there are at least three transaction systems in between (manufacturer to wholesaler, wholesaler to the pharmacy, and pharmacy to the patient), leading to variations of drug pricing in the U.S.

COVID-19 pandemic has highlighted the treatment gaps in the mental health care system. Among these gaps, one can name insurance barriers, financial resources, shortages in mental and behavioral health providers, emergency rooms overload, insufficient beds to hospitalize severely mentally ill patients, uncoordinated mental health providers networks, tele-mental health implementation barriers, as well as disparities to access to care in low-income population and communities of color. A recent report by the Center for the Study of Latino Health and Culture (CESLAC) shows Latinos and Native and black Americans are disproportionately affected by a higher rate of Covid 19 infection, hospitalization, and death (21, 40).

### Emergence of New Neuropsichiatric Disorders

It has been reported that 4 weeks from the onset of COVID-19 viral illness, some post-acute residual effects, complications, or persistent symptoms affect different organs and systems of the body (26). The post-acute COVID-19 syndrome involves the neuropsychiatric system by immune dysregulation, inflammation, accumulation of memory T cells, neuronal injury, dysfunctional lymphatic drainage, microvascular thrombosis, iatrogenic effects of medications, or psychosocial impacts of COVID-19 (26).

Studies have reported that 30–40% of COVID-19 survivors presented anxiety, fatigue, psychological distress, depression, sleep abnormalities, and PTSD after the acute phase of COVID-19 (26). It is recommended that survivors of COVID-19 at high risk for post-acute COVID-19, including those with severe illness during COVID-19 acute phase, admission to ICU, advanced age, or presence of comorbidities, be given integrated outpatient care including screening for neuropsychiatric impairments, neuropsychological evaluation, and imaging studies (26).

## Opportunities to Improve Mental Health Care

Mental health care should concern government and public health advocates to assess and develop better policies and response programs to overcome these gaps and challenges. Policy decisions should focus on strengthening community-based care, support, building local public mental health research capacity, and easy access to treatment and health care services.

Integrating mental health care into the primary care setting could provide a more comprehensive approach to health care following the bio-psycho-social model, enabling early detection, treatment, and accessibility to psychiatric care, minimizing the stigma associated with seeking psychiatric care. The integration process needs adequate financial resources and specialized education and training for primary health care providers (27).

As an approach to address the funding shortages in different mental health sectors, a transfer of funds between different departments could provide a feasible solution (23). The federal government could efficiently address this gap and step forward in fixing the mental health system by removing the Medicaid restriction of funding the patients with mental health issues only in hospitals (28).

Strengthening acts such as the ACA can fill some gaps in the quality and coverage policies by improving access to mental health coverage and addiction treatment. ACA improved preventive care coverage, including screening for depression and alcohol misuse, autism, and behavioral assessments for children. Similarly, ACA expanded the coverage of the essential health benefits involving mental health, substance abuse, and prescription drugs.

Another important action is to increase mental care access and affordability by creating a single-payer healthcare system rather than multiple competing health insurance companies. Due to the lack of transparency and cost control, it is necessary to establish control measures to address this problem. Government and the states should address the cost control of interventions and prescription drugs available to the population (29, 30).

To address mental health during this pandemic, other resources should develop to provide responses and support. Many hotlines such as the National Suicide prevention lifeline, disaster distress helpline, crisis text line, national domestic violence hotline, and the partnership for drug-free kids' helpline can be used as immediate responses. Additionally, financial support resources should be available to relieve financial stress and address its adverse outcomes over mental health.

Evidence-based interventions, such as the Friendship Bench, consisting of a task-sharing approach and implementing a validated assessment tool delivered by trained personnel for early detection and early therapy of mental health problems, are a great way to bridge the mental health treatment gaps (31, 32). Furthermore, implementing early screening and detection of mental illness plays an essential role in improving the mental health system (26). Some actions, such as increasing access to suicide hotlines, are essential to decrease the burden of mental health problems, especially in the Post Covid 19 era.

Continuous evaluation and inter-agency coordination of social welfare programs, such as those addressing homelessness, are deemed to improve mental health and are instrumental in accomplishing the government's strategic planning goals and standards. Coordination of severe mental illness programs requires specific legislation and an appropriate leadership level to achieve successful management (23).

COVID-19 pandemic raises the concerns of a health system with a growing shortage of trained personnel. It is difficult to incentivize mental health professionals to work in areas with limited resources and state licensure regulations.

The U.S. medical graduates are not enough to face the weaknesses of a challenged mental health system. This shortage could be quickly addressed by facilitating licensing for international medical graduates, enabling across-state licensing, and increasing access to trained international medical graduates to areas of need (24, 25). Telemedicine and other digital tools can tackle the barriers in mental health care delivery, improve access, affordability, and shortages of services, especially in the post-COVID 19 era (33). Recommendations to improve the burden of mental illnesses in the post-COVID era are mention on Table 1.

**Table 1:**
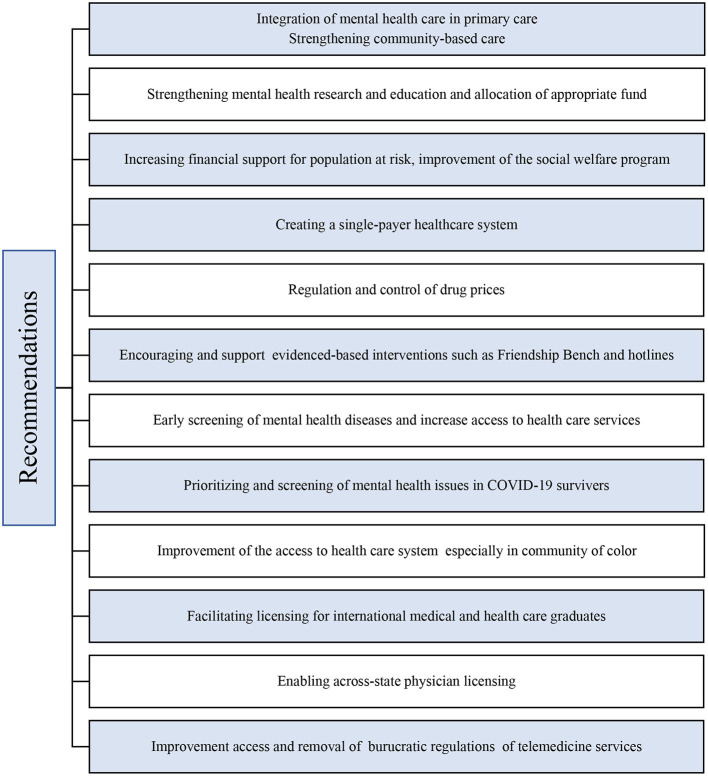
Recommendations to improve the burden of mental illnesses in the post-COVID era (22–34).

## Conclusion

Mental illness has been a public health problem for a long time due to its high prevalence. Public mental health services significantly deteriorated over the past three decades, suggesting that a fair amount of mental health care funds is lost to fraud, excess profits, or are wasted, rather than being used toward mental health care (38).

Delivering high-quality mental care to the U.S. population has been challenged due to the numerous gaps in the mental health care system such as treatment disparities, high drug pricing, uncoordinated systems, failure of effective policies, structural issues, workforce shortages, lack of funding, inaccessibility, and financial barriers. Addressing these gaps and challenges is more alarming nowadays due to increasing mental health issues caused by the COVID-19 pandemic.

The mental health care system needs to evolve and be integrated with primary care. By strengthening the leadership and governance for mental health, reducing mental health stigma, assuring the provision of adequate integrated mental health and social community-based services, implementing strategies for promotion, prevention, and early detection, implementing e-health, increasing trained personnel, and strengthening the information systems, the U.S. federal government could overcome current challenges, assist the delivery of adequate mental health and social well-being to its citizens and eliminate disparities. It is important to educate health care workers to monitor and support mental health needs. Also, there is an increased need for research to assess psychological factors, understand causal mechanisms, and propose interventions to improve mental health. The findings of this manuscript could help in service planning and identification of research priorities.

## Author Contributions

AR made contributions to conception, design of the work, acquisition, and interpretation of the findings, has drafted the work, and substantially revised it. MF and JS contributed to data acquisition, interpretation of findings, drafted the work, and substantially revised the manuscript. IF supervised the acquisition, interpretation of findings and drafted the work, and substantially revised the manuscript. All authors approved the final submitted version, agreed to be personally accountable for its own contributions and ensures that questions related to the accuracy or integrity of any part of the work, even ones in which the author was not personally involved, are appropriately investigated, resolved, and the resolution documented in the literature, and agreed with the order of authors.

## Conflict of Interest

The authors declare that the research was conducted in the absence of any commercial or financial relationships that could be construed as a potential conflict of interest.
